# ‘Get your own house in order’: Qualitative dialogue groups with nonvaccinating parents on how measles outbreaks in their community should be managed

**DOI:** 10.1111/hex.13511

**Published:** 2022-05-12

**Authors:** Kerrie Wiley, Penelope Robinson, Chris Degeling, Paul Ward, Julie Leask, Stacy Carter

**Affiliations:** ^1^ Sydney School of Public Health, Faculty of Medicine and Health The University of Sydney Sydney Australia; ^2^ Australian Centre for Health Engagement, Evidence and Values (ACHEEV) The University of Wollongong Wollongong Australia; ^3^ Faculty of Health Torrens University Adelaide Australia; ^4^ Susan Wakil School of Nursing and Midwifery The University of Sydney Sydney Australia

**Keywords:** childhood vaccination, deliberative methods, outbreak management, qualitative methods, vaccine rejection

## Abstract

**Objective:**

Communities with high levels of vaccine rejection present unique challenges to vaccine‐preventable disease outbreak management. We sought perspectives of nonvaccinating parents to inform public health responses in such communities.

**Methods:**

Nineteen purposively sampled nonvaccinating Australian parents participated in one of seven online dialogue groups. We asked what they thought parents, school principals and public health professionals should do in a hypothetical school measles outbreak and used a framework approach to data analysis.

**Results:**

Parents' views were grounded in strong beliefs in parental responsibility and the belief that vaccines are not effective, thus unvaccinated children do not therefore pose a threat. They then reasoned that the forced exclusion of unvaccinated children from school in a measles outbreak was disproportionate to the risk they pose, and their child's right to education should not be overridden. Nonvaccinating parents judged that all parents should keep sick children at home regardless of disease or vaccination status; that school principals should communicate directly with parents and avoid using social media; that public health professionals should provide information to parents so they can decide for themselves about excluding their children from school; that public health responses should avoid accidental identification of unvaccinated children and that mainstream media should be avoided as a communication tool.

**Conclusion:**

Nonvaccinating parents do not always agree with current Australian approaches to measles outbreak management. Their perspectives can inform approaches to outbreak responses in communities with high levels of vaccine rejection.

**Patient or Public Contribution:**

We sought input from individuals who did and did not vaccinate on study design in its early phases. Individual conversations were used deliberately as we felt the group advisory situation may have felt less safe for nonvaccinating parents, given the divisive and often hostile nature of the topic.

## 1 INTRODUCTION

Community acceptance of vaccination is central to the successful management of vaccine‐preventable disease (VPD), and vaccine rejection is an issue often compounded by geographical clustering.^1^ At a population‐level, Australian childhood vaccine coverage is high and stable with 94.2% of 5‐year‐old children fully vaccinated. However, when the data are stratified into geographical areas, vaccine coverage falls to as low as 78% in some areas,^2^ presenting challenges for public health professionals in managing VPD outbreaks in these localities.

Outbreaks such as measles require rapid public health responses to identify and isolate cases and contacts and identify the vaccination status of exposed individuals. Broader public health interventions such as school closures may also be needed. This requires active engagement with all members of the community, including those whose children are not vaccinated. We sought to understand the perspectives of vaccine‐refusing parents who are currently subject to mandatory policies,^3^ and what they feel should and should not occur as part of the public health response during a VPD outbreak in the community.

Measles is a highly contagious viral disease with potentially severe respiratory and neurological complications, including pneumonia, encephalitis and death. Approximately 30% of measles cases have been reported to have complications.^4^ Global measles incidence increased from a historical low of 18 cases per million population in 2016 to 120 per million population in 2019^5^ due to gaps in vaccination coverage,^6^ and global health agencies are now warning that delays in routine immunization programmes due to COVID could lead to another measles resurgence in the near future.^7^, ^8^

In Australia, state health departments manage infectious disease outbreaks. While the detailed wording and caveats differ slightly between states, the public health response generally follows the national guidelines developed by the Communicable Diseases Network of Australia.^9^ For outbreak management, persons considered to be at risk of measles infection include those who have not previously had measles and those who have not received two doses of measles‐containing vaccine. Most state public health acts require that a child's vaccination records be provided on enrolment in school and make provision for unvaccinated children to be excluded in the case of a VPD outbreak such as measles. Unvaccinated or partially vaccinated children are currently banned from daycare facilities in five of Australia's seven states and territories. Our previous work explored public health professionals' priorities and needs during a VPD outbreak in a low‐vaccination community. It identified their main concern as the threat of a large outbreak, followed by prioritizing isolation of cases and protecting the vulnerable members of the community.^10^

Given the role of public compliance with things like isolation orders in outbreak management, understanding and incorporating the views of vaccine‐refusers into the public health response is crucial to success. This study is part of a broader investigation into understanding childhood vaccine refusal in the context of public health policy, building on the findings of a qualitative interview study to understand their lived experience of vaccine refusal.^11^, ^12^ Here we aimed to understand nonvaccinating parents' values and reasoning on how they think measles outbreaks should be managed and how various implicated actors in such outbreaks should act in outbreak management.

## 2 METHODS

Dialogue group methodology supports conversations between community members about the moral and ethical dimensions of an issue. They differ from standard focus groups, in that they seek to move beyond mere descriptions of people's attitudes and opinions, uncovering reasons for *why* people hold those views and have been successfully used to explore the reasons underlying people's views on difficult topics.^13^, ^14^

We purposively sought parents who had refused some or all vaccines for their children. We contacted previous participants in our grounded theory study of nonvaccination.^11^ We also approached Facebook parenting groups individually in geographical areas known to have high levels of vaccine rejection and advertised more broadly via Facebook, targeting Australian parents with children under the age of 18. Online groups were chosen to bring together people from a variety of geographical regions and to increase convenience and anonymity for participants. Recruitment continued until saturation was reached, and no new reasons for views on how a measles outbreak should be handled were given. All dialogue groups were audio‐recorded and transcribed professionally. All participants were assigned pseudonyms and all potentially identifying information was removed from the transcripts. The polarized nature of vaccination‐related discourse in Australia meant that some participants were unwilling to provide any demographic detail beyond their first name and state of residence, for fear of being identified.

All participants were given a participant information statement outlining the study, and participants gave written informed consent and were compensated for their time with an AU$80 gift voucher. This substudy was approved by the University of Wollongong Human Research Ethics Committee, approval number 2019/244.

For a focused discussion, participants were presented with a hypothetical scenario set in a small community with a number of families who refuse vaccines (see Box 1 and Supporting Information Material). As the conversation progressed, we introduced complicating factors to the scenario, such as an immunocompromised child in the class. Participants were asked to respond to the scenarios where they felt the main stakeholders—parents, the school and public health practitioners—should act; participants were also asked to explain the reasons underlying these normative judgements (Box 2).

BOX 1 **Summary^a^ of scenarios posed to the dialogue groups for discussion**

**Table:** 

**Hypothetical setting**
A small town with a high level of vaccine rejection in the community. The town has a single primary school where about 70% of the students there are fully vaccinated. The three main actors in the scenario are the public health officer responsible for outbreak management; a mother of two unvaccinated children, one of which attends the school; and the school principal.
**Questions posed to participants**
The participants were progressively given the following scenarios and asked what they thought each actor should do at a number of timepoints.
They were also asked what they think members of society owe one another, and what they think is reasonable for society to expect of parents in relation to vaccination.
**Scenario 1**
*Wednesday*: The public health officer receives a report of a suspected case of measles in a local primary school child.
*Friday*: Five children from the school have a confirmed measles diagnosis, three from one class and one each from two other classes. The school principal contacts all parents advising of a measles outbreak at the school. Parents of children in the affected classes get a separate additional communication that the public health unit will be in touch shortly.
*Friday–Saturday*: Public health staff talk to about 70 parents, including the mother of two unvaccinated children, advising that their child may have contracted measles at school. Parents are advised that unvaccinated children are considered to be a susceptible contact and ask that such children be kept home from school and monitor for symptoms. Once no more cases of measles appear at the school, they will count 14 days, then unvaccinated children will be able to go back to school.
**Scenario 2**
*Saturday–Sunday*: The public health officer learns that the confirmed cases have visited several venues. He organizes a vaccination clinic at the school for parents who would like to vaccinate their children, alerts local media, and provides the school principal with a list of children who have been asked to be kept at home. The school principal uses the school Facebook page to ask parents to keep their children at home if asked to, and advising that the vaccination clinic will be available, and consent notes will be sent home. The comments on the Facebook page vary: Some parents have commented positively on the post. Others are angry, confronting nonvaccinating parents. Yet others are blaming the school for over‐reacting to a normal minor childhood illness, or expressing fear that children might be vaccinated without consent from their parents.
Ten more cases of measles are identified by Sunday afternoon.
*Monday*: The school principal advises the public health officer that some children with symptoms have come to school and asks for advice.
**Scenario 3**
A child at the school has leukaemia.
**Scenario 4**
The mother keeps her child home as requested, and after a few days she becomes unwell. The GP suggests strategies for treating the child and keeping her isolated, and asks the mother whether she might be willing to vaccinate her other unvaccinated child.

a For the full scenario as presented to the participants, see Supporting Information Material.

BOX 2 **Guiding principles for effective engagement with nonvaccinating parents during an outbreak, with practical recommendations**

**Table:** 

1. **Acknowledge the right of nonvaccinated children to education**
⇨ Ensure school exclusion periods are as short as possible, and all efforts are made to continue to include all children in learning throughout an outbreak.
⇨ Systems and resources are required to make e‐learning options for excluded children equitably accessible to all school communities.
⇨ Parents will tolerate short, well‐justified periods of exclusion of their children from school, but are unlikely to believe these are justified. If the exclusion is being considered, ensure that it is epidemiologically justified.
2. **Protect the privacy of all children and families in their communities**
⇨ Care should be taken to ensure that activities in response to outbreaks do not accidentally identify unvaccinated children and their families in the community.
3. **Engage with all parents and children with respect**
⇨ Avoid using communication channels that can amplify or encourage disrespectful communication (e.g., social media, unless it is heavily moderated or comments disabled).
⇨ Parents should be contacted discreetly, treated respectfully, and given information, but not pressured to vaccinate.
⇨ Mainstream media could be used to disseminate information about the outbreak and public health needs, but care needs to be taken to avoid inflammatory framing and focus on vaccine rejection.
4. **Recognize the often‐substantial efforts of nonvaccinating parents to support their children to flourish**

Framework analysis methodology was used to analyse the discussions.^15^ A coding frame based on the research questions was developed and three researchers independently coded one transcript against it. The respective coding was compared and discussed, and some extra inductively arising themes were added to the framework, which the remaining transcripts were coded against by two of the researchers.

We approach this study with a public health orientation accepting the overwhelming evidence that the benefits of vaccination far outweigh the risks. However, we also see value in understanding the beliefs and behaviours of nonvaccinating parents, and acknowledge the tension between the two positions. Reflexivity was maintained through debrief discussions held after each dialogue group, and subsequent discussions during the analysis and reporting phases, which allowed us to remain conscious of our own positions as researchers, and our role in the research throughout the entire process.

## 3 RESULTS

Seven 2‐hour online dialogue groups with 19 participants were conducted between November 2019 and July 2020. One discussion was conducted as an interview, as one of the two participants dropped out of the call due to technical difficulties. All participants were women, 18 of whom lived in one of five of Australia's eight states and territories, and one who did not wish to divulge her state of residence. The participants had children ranging from under 5 years old to young adults (Table 1).

**Table 1:** Dialogue group participant structure and date

Dialogue group	Date	Participant pseudonyms and state of residence
Group 1	13 November 2019	Marika (New South Wales)
		Rachel (Victoria)
		Emily (New South Wales)
		Danielle (Western Australia)
Group 2	13 December 2019	Anita (New South Wales)
		Sharon (Victoria)^a^
Group 3	16 December 2019	Maria (New South Wales)
		Frances (New South Wales)
		Rosemary (South Australia)
		Jaclyn (South Australia)
Group 4	5 June 2020	Francine (South Australia)
		Sally (South Australia)
		Ellie (New South Wales)
Group 5	19 June 2020	Alex (New South Wales)
		Martina (Queensland)
Group 6	26 June 2020	Annaliese (state not given)
		Jacinta (Queensland)
Group 7	1 July 2020	Roberta (Queensland)
		Leanne (Queensland)

^a^ Participant dropped out due to technical difficulties.

**Figure 1:**
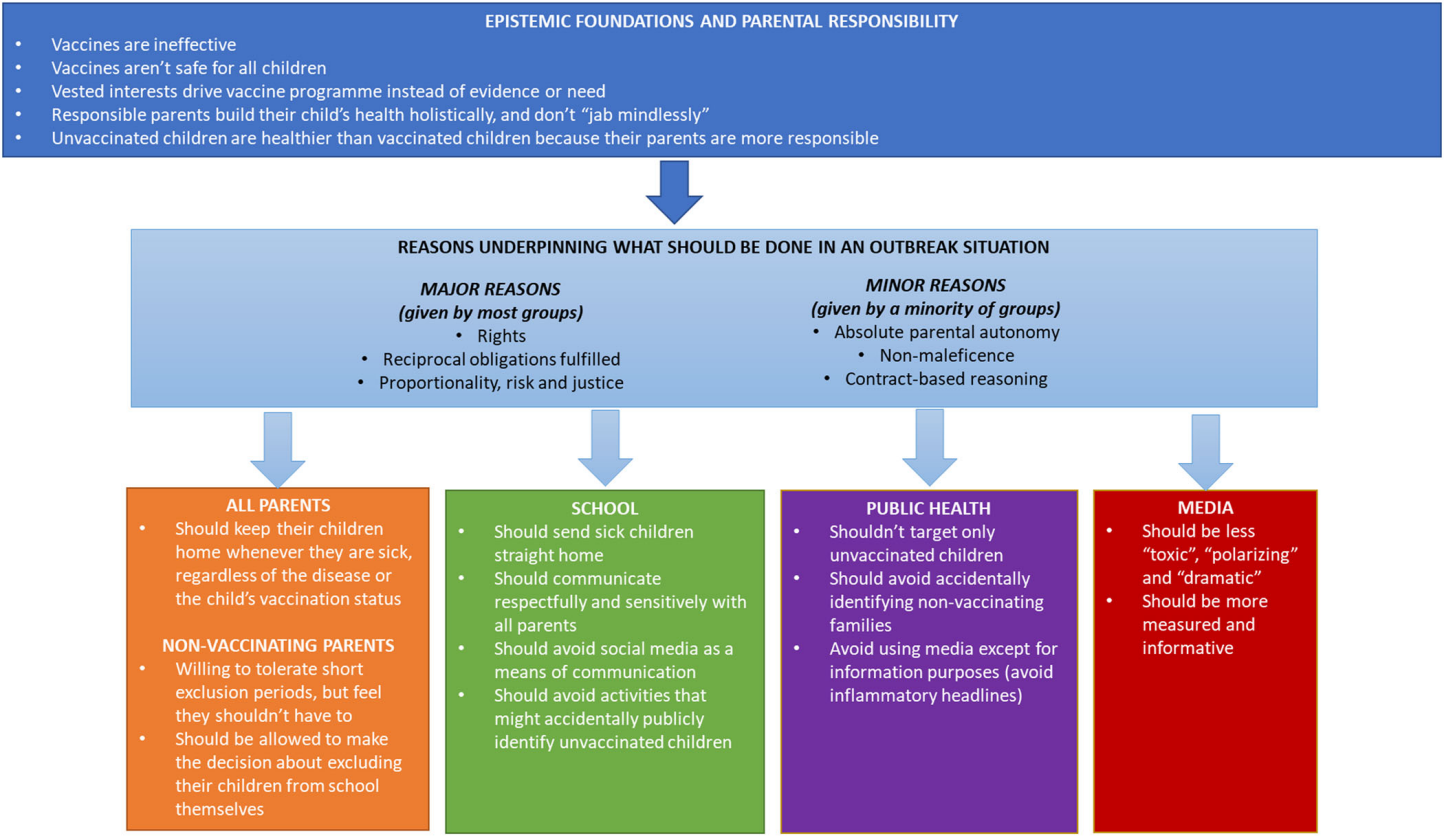
Nonvaccinating parent's views on what community different stakeholders should and should not do when a measles outbreak occurs at a local school, including common reasons

We begin with the context in which the parents view vaccination and parenting practices. We then report the reasons they relied on in making judgements on how measles outbreaks should be managed in a small community with high vaccine rejection. We then report parents' views on how they think the various actors—parents, the school, public health professionals and the media—should act in such a scenario, and the reasoning given for these views. Finally, we discuss how these findings can inform outbreak management where there is high community resistance to vaccination (see Figure 1).

### 3.1 Contextual issues

To understand parents' reasoning about measles outbreaks, two underpinning issues need to be understood: the participant's epistemic foundations for their positions (basic beliefs about what could be considered true with respect to vaccination) and their general views on parental responsibility.

#### 3.1.1 Epistemic foundations of participants' arguments

Participants shared beliefs that are common among vaccine‐rejecting parents, which have previously been reported in Australian research.^11^, ^12^, ^16^, ^17^, ^18^ We refer to these as epistemic foundations because they were basic beliefs about what could be considered true in respect of vaccination, and because they underpinned other beliefs (e.g., normative positions regarding the right course of action). Participants broadly believed that vaccines do not prevent disease and that vaccine‐preventable diseases are not generally dangerous. A few believed measles to be of little consequence, and a small number believed it to be beneficial. A small number of participants believed their child had suffered an adverse event following immunization; a minority of these believed that vaccinations generally work, but that their children were especially vulnerable to severe reactions to vaccines. These parents did not reject vaccines as such, but rejected vaccines for their children, and were more likely to believe that diseases like measles are of concern. Participants did not reject vaccination science wholly: rather, they made complex arguments about deficiencies in the science, including the perceived role of ideology and vested interests on the part of policy decision‐makers and vaccine manufacturers, with resulting effects on the state of vaccine evidence. As such beliefs have already been reported in communities of nonvaccinating parents^11^, ^16^, ^17^, ^18^, ^19^, ^20^, ^21^, ^22^ we will not describe them further, but they should be held in mind as an important backdrop to the findings below.

#### 3.1.2 Participants' views on parental responsibility and practice

Within and between groups, participants shared strong views on parental responsibility, and the level of responsibility taken by vaccinating and nonvaccinating parents.

Participants broadly agreed that nonvaccinating parents take better care of their children than vaccinating parents, who ‘just jab mindlessly’ (Jacinta, Group 6) and do not put thought or work into their children's health. In this view, vaccinating parents were seen as careless about infectious diseases, mistakenly believing their children are safe from all infections due to their vaccination status:

*[P]eople who are vaccinated also need to be a little less blasé in terms of yes, we're vaccinated but we are not therefore immune. And I know numerous families who have sick children everywhere passing on their sickness and they feel 100% assured because it can't be serious because they've had their jabs*. Danielle, Group 1

Participants shared a strong view that unvaccinated children are healthier than their vaccinated peers. For some, vaccinated children were the problem (as opposed to unvaccinated children) as they are not robust enough to withstand disease because of their parent's practices:

*If people would stop being so afraid of somebody infecting them with something, and realize it's not the pathogen, it's where it falls. Get your own house in order*. Annaliese, Group 6

In one of the groups, all participants had refused vaccines because they believed their children had experienced adverse events following immunization. Only this group did not reason from a belief that vaccinating parents were irresponsible.

With these two shared contextual factors—epistemic foundations and parental responsibility—as underlying assumptions, we now turn to parents' reasoning about outbreak management.

### 3.2 Nonvaccinating parents' judgements about managing measles outbreaks

We identified four main lines of reasoning parents used to make a range of judgments on how they felt a measles outbreak should be managed in a school with a known group of unvaccinated children (refer to Figure 1 and Table 2). These lines of reasoning were not mutually exclusive and occurred in different combinations among the groups.

**Table 2:** Reasons that nonvaccinating parents relied upon in making judgements about managing measles outbreaks

Reasons and judgements with respect to outbreak management *As described by nonvaccinating parents*	Actors	
	*Parents (nonvaccinating and vaccinating)*	*Public health, schools and media*
**Major reasons (given by most groups of nonvaccinating parents)**		
**Rights**	Parents should be able to send healthy children to school for education, regardless of vaccination status.	*Right to education*
*Right to education*		
*Children enjoy a universal right to education, and this should not be abrogated*.		The health of other children should not outweigh the rights of unvaccinated children to access education.
*Right to respect*		*Right to respect*
*All parents and children deserve to be treated with respect*.		All communication from schools and public health should be respectful.
*Right to confidentiality and privacy*		Communications should be directed at all parents, not just those who do not vaccinate.
*Parents and children should retain control of information about their vaccination status, and this should be kept confidential by others*.		Communication should be via channels that do not invite public comment and online harassment (e.g., direct communication with parents, rather than using Facebook and other social media platforms).
		Media coverage should be less polarizing and less focused on the unvaccinated. Instead, focus should be on unbiased information about the outbreak.
		Some felt the media should be kept out of it altogether.
		*Right to privacy*
		Excluding unvaccinated children will identify them as unvaccinated to the community; therefore, exclusion should not focus on them.
		Onsite vaccination clinics at the school should be avoided to avoid the public identification of nonvaccinating families.
**Reciprocal obligations fulfilled**	Nonvaccinating parents should be able to send their unvaccinated children to school because they fulfil their reciprocal obligations to the rest of the community by taking better care of their children.	Public health, schools and media should not view nonvaccinating parents as ‘owing’ anything to society, and therefore not use this as justification for excluding unvaccinated children from education.
*Nonvaccinating parents go to such lengths to raise healthy children that they do not owe anything further to society*.		
**Proportionality, risk and fairness**	All parents should keep symptomatic children at home, or immediately collect them from school should they become ill, regardless of vaccination status or the disease.	Schools should immediately send any sick child home, regardless of disease or vaccination status.
*Decision‐makers focus disproportionately on unvaccinated children, these children are denied education access when the risk they pose is small, and this is unfair to unvaccinated children*.		Healthy, unvaccinated children should not be excluded, as they do not pose a risk.
		Immunocompromised/vulnerable children should be excluded as they are vulnerable to everything, and their right to education should not override a healthy unvaccinated child's right to education.
**Minor reasons (given by a minority of groups of nonvaccinating parents)**		
**Absolute parental autonomy**	Parents are responsible for making decisions for their children and have the right to choose whether to send their child to school or not, based on their individual situation.	Schools and public health should provide information to parents to enable them to decide for themselves whether to send their child to school.
*Only parents should have the power to make decisions in their children's interests because this is an inviolable right for both children and parents*.		
**Nonmaleficence**	Parents should not have to choose between believed harm of their child through vaccination and denying their child access to education.	Public health should consider children who are vulnerable to vaccine side effects in policy decision‐making.
*Vaccines harm children, so I should not be forced to vaccinate my child*.		
**Contract‐based reasoning**	Parents who choose not to vaccinate often do so knowing that their child will likely be excluded from school/daycare in the event of an outbreak. While they disagree, some parents are willing to accept short exclusions. Most, however, feel their healthy children should be allowed to attend at their parent's discretion.	
*Parents formally agree to certain conditions when enroling children at school, so are bound by these conditions*.		

3.2.1

1. *Rights, reciprocity and proportionality*

Most groups endorsed three interconnected lines of reasoning around *rights*, *reciprocal obligations fulfilled* and *proportionality, risk and fairness*.

1a) *Rights*

Participants frequently used human rights discourse to justify their positions. This included reference to formally recognized universal human rights (e.g., the right to education, the right to privacy), as well as less formal use of rights discourse, such as the right to being treated with respect and the right to choose freely.

Children's right to education was a recurring theme across all groups. Participants asserted strongly that children have a right to education, regardless of their vaccination status; some used this to argue that exclusion was never justifiable.

*Exclusion to education is no answer. I'm not disagreeing that if you're symptoming you should be allowed to continue at school like regular, but when you are completely excluding a child from the opportunities that happen in a* [school or preschool] *environment,…* [it's] *totally unacceptable*. Frances, Group 3

Some objected to the derogation of the right to education in service of public health goals:

*Education is a human right, and you work to collaborate with the parent for health outcomes and education outcomes. One doesn't supersede the other*. Roberta, Group 7

The right to privacy was also a recurring theme, often grounded in previous experiences. Of particular concern was that in‐school vaccination programmes could accidentally identify unvaccinated children to the broader community. Experiences with unwanted identification were particularly important in arguments about how schools should handle outbreaks regarding the exclusion of only unvaccinated children.

*The program was rolled out at school and all of the girls at that age were getting the vaccine. We did not sign a consent form … it had ramifications … a small rural community. Everybody knew … when you offer something in a school, you do totally expose anybody that has a different perspective*. Frances, Group 3

1b) *Reciprocal obligations fulfilled*

When specifically asked what they feel is reasonable for society to expect of nonvaccinating parents, the participants' response was generally that nonvaccinating parents do not owe society anything because of the lengths they already go to in raising healthy children who can withstand the diseases that vaccines are intended for:

*The way I see it is that … parents who choose not to vaccinate their children do actually offer quite a lot in return. They choose to breast feed their children longer, they choose to minimise sugar and processed foods and they choose to keep their kids as healthy as they can so that, when they do contract an infection, then obviously their symptoms are going to be less… they generally keep their kids home when they're sick… These decisions to not vaccinate are not made in a blasé way and they really work hard to keep their kids home, look after them properly and ensure that they're disease doesn't progress to something that's dangerous. And in doing so they're protecting the population*. Martina, Group 5

Given the level of responsibility they felt they took as parents, participants felt they did not owe anything further to the community and should be allowed to decide whether their child attends school on their individual situation.

1c) *Proportionality, risk and fairness*

Parents believed that decision‐makers focus disproportionately on unvaccinated children as those children pose little if any risk to the community. There was some suggestion that current approaches disproportionately treat unvaccinated children as the only threat to other children:

*I think it's… remove the unvaccinated because there's an outbreak, because they're dangerous, but once you remove them, they are not at school, then the school is completely safe*. Emily, Group 1

Several other reasons were given to challenge the exclusion of unvaccinated children from school. These included: (1) that the measles vaccine was not effective, and so vaccinated children carried similar risks to unvaccinated children; (2) that there was a disproportionate focus on measles with no contact tracing and isolation for other viral diseases or (3) that unvaccinated children may have had a prior infection and may therefore be immune, and so should not be excluded. These positions were sometimes argued in relation to the evidence underpinning public health approaches:

*[T]he unvaccinated or partially vaccinated kids are perceived as being a higher risk to other people and to themselves. And so they're removed … But I don't necessarily think that there's enough reasonable evidence to assume that those kids are going to be more of a risk …The vaccination doesn't stop the other children from transmitting the disease to other people*. Sally, Group 4

Participants saw framing unvaccinated children as a threat as misguided and unjustified; this arose particularly in the context of tradeoffs between different groups of children. For example, when the issue of an immunocompromised child at the school who is unable to be vaccinated was raised as a scenario, one participant framed it in terms of inequities:

*I guess you're trying to compare the inequity for a sick kid versus the inequity for an unvaccinated kid. I think the difference for the unvaccinated kid is there's no proof that that child is a greater threat or anything to other children*. Ellie, Group 4

Others agreed with this sentiment: immunocompromised children were seen as vulnerable to everything. The extremity of their vulnerability put limits on what accommodations could be reasonably expected from other families. Participants reasoned that vaccinating their own children entailed a personal risk, and they should not be required to take this risk: The extreme vulnerability of immunocompromised children meant that it was not reasonable to hold other parents and children responsible for their protection.

*I find that the ethics around getting the vaccine to prevent someone else from getting it, to protect some vulnerable person, that is really unethical … what you're saying is take a risk with your health to protect a vulnerable person. And the reality is that vulnerable people are vulnerable to everything, they're not just vulnerable to the diseases that we have a vaccine for; they're vulnerable to all infections… it is extremely unethical to be coercing people into vaccinating themselves or their children in order to protect vulnerable people*. Martina, Group 5

3.2.2

2. *Absolute parental autonomy*

A minority of participants held that parents are responsible for making decisions for their own children, that they should not be forced to do anything, and that it was up to parents to decide whether to isolate their unvaccinated child from school in the case of an outbreak.

*We should have more autonomy for parents to make decisions … trusting that those parents are making the right choices for their children based on the knowledge that they have*. Sally, Group 4

3.2.3

3. *Nonmaleficence*

A small number of parents felt that their children were especially vulnerable to vaccine side effects and that this was not being considered in public health policy. They felt they were being unreasonably asked to choose between disabling their own child through vaccinating or risking a mostly nonfatal disease. When their child's right to education was also threatened through exclusion from school, parents felt they were being asked to make impossible choices.

*I was told that my son's encephalitis was caused due to the whooping cough component [of the vaccine] … But then his rights to education are threatened. I think that's discrimination. I shouldn't have to choose between [his education and] … him being permanently disabled*. Roberta, Group 7

3.2.4

4. *Contract‐based reasoning*

A minority of participants suggested that nonvaccinating parents did have an obligation to exclude their children from school during an outbreak. This was justified in relation to an implicit or explicit contract that the parents had entered when enroling their children—usually a contract with the school or day care facility. Danielle and Maryke, for example, drew on personal experience of having their child excluded from childcare due to pertussis outbreaks, conceding that nonvaccinating parents should accept the need to quarantine their children in these situations, even if they disagree with it:

*Yeah I agree with Danielle… if you're making this decision of not vaccinating your child, you kind of have to bear the responsibility of the fact that something like this might happen and you've got to act upon it accordingly. So I believe that part of our responsibility is also knowing the symptoms of all the diseases and things like that, so that you can catch it as soon as possible and know how it's treated or how to prevent it in different ways than vaccination…*. Maryke, Group 1

Others pointed out that it is the policy of most schools that unvaccinated children must be excluded in an outbreak: Thus ‘it's what you sign up for’ when deciding to forego vaccines and enrol children in school (Frances, Group 3). Even when temporary exclusion was considered tolerable as a form of contractual compliance, it typically was not considered justified on any other principle, especially if it adversely impacted a child's education.

*[P]ersonally I can live with temporary exclusion for unvaccinated children; I can live with that. I don't necessarily agree with it, but I can live with it*. Alex, Group 5

### 3.3 Parent's judgements about what should be done to manage a measles outbreak

Having laid out the contextual factors and underlying reasoning parents used to justify their views on how a measles outbreak should be managed in the community, we now report participants' judgements on how the different actors in the scenario should manage the outbreak.

#### 3.3.1 Judgements about what parents should do in an outbreak situation

In keeping with the core position on parental responsibility, there was general agreement between and within the groups that *all* parents should be aware of the signs and symptoms of diseases, and should keep their children home when sick, regardless of the disease or children's vaccination status. This was sometimes framed in terms of what is best for the individual child:

*[A] parent's job is to nurse their kids back to wellness and there's no place like home, in your room, in your bed, and your favourite things, and a parent that loves and protects you, and cares for you, and listens to you, and let [the disease] run its course*. Roberta, Group 7

Others emphasized responsibility to others:

*[Y]ou might be fine, but the Granny down the road might get bumped off … I've always said this to my kids ‐ it is the responsibility that you've got to not infect other people … Slack parents send sick kids to school, and that is a problem. No kid should be at school sick. The kid comes down with symptoms, keep them home, and that should be the case always*. Jacinta, Group 6

None of the participants mentioned the possibility of being infected with measles before symptom onset. Most, like Ellie, focused on the appearance of symptoms as a cue for taking action:

*"I would probably chose to leave my kids in there for a little bit longer and see if they just start to develop symptoms and just monitor them*. Ellie, Group 4

#### 3.3.2 Judgements about what public health, schools and media should do in an outbreak situation

Drawing on the reasoning outlined above, most participants were of a strong view that public health and school responses to outbreaks should not target unvaccinated children alone. Instead, outbreak responses should focus on automatically sending home any children who presented to the school with symptoms.

While there was some acknowledgement that public health policy and guidelines mean that public health professionals are somewhat limited in how they can respond, there was a clear feeling that healthy unvaccinated children should not miss out on educational opportunities, as described above. Most of the participants suggested that public health professionals should supply parents with high‐quality information and leave parents to choose the right course of action for their child, based on their individual circumstances.

*It is the responsibility of the public health authority to make information available to people and to keep people informed. But I think where we stop in this current scenario is then handing over autonomy and responsibility to those families to make the right choice. We're [public health authorities] still a little bit – we like to dictate in that circumstance, here's the information and here's what you should do. Instead of you decide what's best for your family*. Sally, Group 4

Caring for immunocompromised children in a community was often acknowledged to be a difficult problem, although some expressed annoyance with what they perceived to be a typical public health trope that occurred rarely. As most participants believed unvaccinated children were not at risk or a risk to others, the exclusionary policy would be better targeted at the vulnerable:

*I think the safest way is to keep the immunocompromised child at home while there's an outbreak, rather than keeping the unvaccinated at home because the unvaccinated aren't necessarily sick*. Martina, Group 5

For parents who believed their children had been vaccine‐injured, public health responses appeared to prioritize the rights of the immunocompromised child over the rights of their own child, which they saw as unjustified:

*“I want to do the right thing for the other little child, or other boy, whoever is immuno‐compromised, but my child can't stay home half the year because the additives in this product [vaccine] haven't been investigated. That's equally unethical and equally unjust*. Roberta, Group 7

When asked how authorities and the media should interact with nonvaccinating families, participants routinely emphasized a need for respectful communication, and acknowledgement of parents' agency and autonomy:

*It's always an invitation. It's always an invitation to collaborate. It's always an invitation to discuss … no assumptions, and no generalisations, no enforcing, and no ‘you should’, and no ‘you oughts*’. Roberta, Group 7

Participants suggested direct communication between the school nurse and the parent in the case of an ill child, and direct communication between the school and parents through newsletters and, where required, confidential one‐on‐one conversations with affected parents or individualized text messages sent to parents with children in affected classes. Participants preferred communications that accepted a parent's right to choose and avoided blaming the children themselves, as well as a clear description of what symptoms to look out for in all children, not just those who are not vaccinated.

The right to privacy underpinned parents' concerns about how the public health response may inadvertently identify unvaccinated children and their families to the broader community, which would then put the child and their family at risk of social ostracism. As a mode of communication, there was consensus that schools should not communicate via social media platforms such as Facebook, as this typically leads to heated discussion online:

*“And I think it's a really slippery slope when you start to put this kind of thing on a school social media page. If the policy … is that they need to contact all parents, then I think it's important that they have systems in place that don't rely on social media. Because then you remove that element of vilification – easy vilification where unfortunately social media can become quite awful*. Frances, Group 3

Parents were mixed on whether vaccination clinics should be set up at school. Their main concerns were the potential for coercion of children or violation of privacy. Anita felt it was reasonable for a parent to be offered the choice, so long as the parent's autonomy was respected,

*I think it's reasonable for it to be offered… as long as there's no pressure or coercion that's the main thing*. Anita, Group 1

Others expressed concern and even objection to vaccination clinics being set up on school grounds, as to them it represented an opportunity to make unvaccinated children identifiable to others in the community, and a risk that their child may be coerced into being vaccinated without parents' consent. A suggested alternative was that parents be consulted privately and offered the chance to catch up with vaccination through their local GP, rather than via a public clinic.

There was a very strong consensus about the role the media currently play in outbreak scenarios, and the role they should play. Mainstream media were described as ‘toxic’ and ‘bullying’, ‘polarizing’ and using ‘fear‐based campaigning’. There was a strong consensus that reporting should be less dramatic, and more factual and informative. Because of this, participants argued that the media should not be used as an information conduit to the community.

*I think that would be my last point of call, getting the media involved. I think it's better to keep it local and private and respectful because the media sometimes, I think, forgets these are people we are dealing with, and families*. Leanne, Group 2

Aside from rights‐based reasoning, some participants' previous experiences with outbreaks and interactions with other public health programmes drove their opinion on what a public health officer should do in the given scenario. Emily from Group 1 related her experience:

*I received a call from the health department a few months ago as well and it was a really threatening call, like off‐putting definitely. If you are in doubt whether you should vaccinate your child or not, this is not helpful. So the strategies that they are using are not helping*. Emily, Group 1

Anita, on the other hand, had positive experiences. She is impressed by the way the principal of her child's current school handles communication with parents, limiting social media use and therefore limiting the opportunity for disrespectful online behaviour.

*I know at our school … the principal's been quite clear about what the Facebook page is there for and what it isn't there for. I think he's said having direct conversations between parents and staff members is the best way forward, and concerns should be raised directly with the school*. Anita, Group 2

## 4 DISCUSSION

This study explored the views of nonvaccinating parents on a hypothetical measles outbreak in an area with high levels of vaccine refusal. They describe what actions they feel should and should not be taken by parents, schools, public health officials and the media, and their rationales. Their epistemic position on vaccination aligns closely with previous research in Australia and more broadly^17^, ^21^, ^23^: parents doubted the safety, effectiveness and necessity of vaccines, instead of placing a premium on a holistic approach to health. Also consistent with previous findings, the participants in this study believed that their unvaccinated children are healthier than their vaccinated peers and that their own parenting practices are superior to those of vaccinating parents.^11^, ^16^, ^18^, ^20^

Participants frequently called on *rights, reciprocity and proportionality* to underpin their arguments. It has been previously identified that vaccine rejectors have a heightened moral preference for the rights of individuals.^24^ This rights‐based reasoning fed into a perceived inequity of their child's exclusion from school during a VPD outbreak, which was compounded for some by their feeling that they go to greater lengths than vaccinating parents to ensure their child's health through holistic care. They felt their unvaccinated children were better equipped to withstand the diseases than their vaccinated peers, and there was therefore little justification to exclude their children in an outbreak. This apparent cultural perception is embedded in a complex combination of social identity^25^, ^26^ and a kind of ‘in‐grouping’ seen as a result of the social stigma applied to them.^12^

*Absolute parental autonomy* was important to a minority of participants, who feared being forced to vaccinate their children. Our research with public health professionals showed that identification and vaccination of unvaccinated children in such a scenario is not, in fact, a priority for decision‐makers, with isolation of cases and control of the outbreak through other means reported as being of higher importance.^10^ Some parents believed they should have autonomy in deciding whether to send their children to school during a measles outbreak, based on their own assessment of the situation. More broadly, participants' views on what parents should do in an outbreak scenario were largely based on the appearance of symptoms, while the possibility for asymptomatic infectiousness was almost exclusively not acknowledged or perhaps not known.

*Nonmaleficence* underpinned some parents' reasoning that they should not be asked to vaccinate (and therefore, in their view, harm) their own children in a bid to protect others. While some parents in our sample did not see measles as a threat, others did acknowledge that minimizing disease spread was what a responsible citizen should do.

Many participants were concerned about potential inadvertent identification through school exclusion of unvaccinated children during an outbreak, based on previous negative experiences. Research has shown that Australian nonvaccinating parents are subject to significant social consequences if their position is made public, suffering social ostracism, online bullying, family rifts and differential treatment in medical settings owing to their vaccine decisions. Therefore, the fear of inadvertent identification described in this study is somewhat justified.^12^

Participants spontaneously identified the media as actors with an agenda, citing the derogatory way in which nonvaccinating parents are often portrayed and the unhelpful ‘frenzy’ that often accompanies reportage on vaccine rejection. Here too, parents grounded their argument on a right to respect, and in many cases based on negative personal experiences.

Our findings can inform meaningful engagement with nonvaccinating communities during VPD outbreaks; however, the core epistemic differences between public health and nonvaccinating approaches means that agreement or even compromise will not always be possible. Balancing parents' prioritization of their child's right to education with the need to minimize disease transmission can be difficult. Recent online learning responses to COVID‐19 could provide a kind of ‘middle ground’ whereby affected children are physically excluded without disadvantaging their education. However, given the inequities experienced during COVID‐19 lockdowns,^27^ resources are required to make this a sustainable solution for all school communities. Our findings suggest that some nonvaccinating parents may still find this differential treatment unjustifiable, believing their holistic parenting practices result in healthier children who can withstand measles. This is more difficult to address, and insights from the depolarization cultural/social change literature may offer useful starting points.

Some parents' preference for autonomy in deciding whether to exclude their child from school during an outbreak suggests approaches appealing to their desire for agency and respect may be helpful. Clear and transparent information shared directly with parents, and two‐way conversations between public health agencies and parents may help build more trusting relationships. Because reasoning from nonmaleficence was highly salient for some parents, it may be possible to encourage them to avoid causing harm through infection during an outbreak by voluntarily isolating if exposed, especially if detailed information is given about infectious but asymptomatic incubation periods. In a post‐COVID context, public awareness of infectious disease transmission may increase the feasibility and potential impact of such messaging.

To allay fears of coercion when implementing in‐school vaccination clinics, the immediate public health priorities (i.e., case isolation and outbreak control, rather than vaccinating unvaccinated children) should be clearly communicated. Furthermore, the unintended consequence of being identified as a nonvaccinator to the rest of the community should be a serious consideration for public health and school stakeholders, and guidance on how to sensitively communicate with, and where needed exclude exposed unvaccinated children from school is needed to protect those families from social harm. We recommend better enforcement of nonattendance policies for sick children as an important first step. The context of COVID may present new opportunities to encourage this as a social norm. Further research on parents' views in the post‐COVID context would be useful to ascertain whether perceptions have subsequently shifted.

Our inductive finding that parents view the media as agenda‐driven actors, which should not be used as communication channels for outbreak management, is unsurprising in light of the often—disparaging framing of nonvaccinating parents by the Australian media.^28^ Research has found that journalists see themselves as conduits of information and as public watchdogs, with differing views on the legitimacy of sensationalist or emotive reporting.^29^ In this light framing nonvaccinating parents as deviant could be seen as a public service on the part of the media; however, research suggests that such reporting contributes to a highly stigmatizing environment that can lead to social harm.^12^ Working with the media to encourage critical and independent reporting of vaccine‐related issues without causing harm is crucial to outbreak management strategy.

This study has some limitations. While efforts were made to recruit nonvaccinating parents with a wide variety of views, the provocative nature of the topic in Australia may have discouraged participants with differing views from participating. While it is possible our online groups may have been somewhat different if held in person, evidence suggests that data quality from the two modalities is comparable.^30^ Our data set included dialogue groups conducted before and after the advent of the COVID‐19 pandemic and the associated large‐scale public health response, which raised the potential for views of the groups to differ, depending on when they were conducted in relation to the unfolding COVID‐19 situation. While later groups did mention COVID‐19 and the applicability of some of our hypothetical scenarios to their real‐life experiences, at the time of the last dialogue group a COVID‐19 vaccine was still not available, and vaccination was not generally a part of the COVID‐19 discourse aside from speculation on when one might be available. As a result, we found that our findings were generally consistent over the course of the study.

## 5 CONCLUSION

A measles outbreak in a community with high levels of vaccine rejection presents a public health challenge for parents, schools and public health agencies. School exclusion of exposed unvaccinated children forms part of Australian public health responses; however, nonvaccinating parents do not always agree with or accept this approach. Our unpacking of the reasons behind nonvaccinating parents' judgements on how such a scenario should be managed suggests that care is needed in how public health risks and remedies are framed and communicated, and how unvaccinated children and their families are dealt with. Focus and resources are needed to assist the media in reporting vaccine‐related issues in a way that does not cause harm and to enable the exclusion of nonimmune children from educational settings when necessary, in a way that does not disadvantage them.

## AUTHOR CONTRIBUTIONS

**Kerrie E Wiley**: Investigation, formal analysis, writing – original draft, writing – review and editing, visualization, project administration. **Penelope Robinson**: Investigation, formal analysis, writing – original draft, writing – review and editing, visualization, project administration. **Chris Degeling**: Conceptualization, methodology, writing – review and editing, funding acquisition. **Paul R Ward**: Conceptualization, methodology, writing – review and editing, funding acquisition. **Julie Leask**: Conceptualization, methodology, writing – review and editing, supervision, funding acquisition. **Stacy M. Carter**: Conceptualization, methodology, formal analysis, writing – review and editing, supervision, funding acquisition.

## CONFLICTS OF INTEREST

The authors declare no conflicts of interest.

## Supporting information

Supporting information.

## Data Availability

The data are not publicly available due to privacy and ethical restrictions. Institutional Review Board conditions of approval for this study state that the data must not be shared with persons not listed on the approved application.
