# Supplementary material for: ‘Get your own house in order’: Qualitative dialogue groups with nonvaccinating parents on how measles outbreaks in their community should be managed
Source: Health Expect. 2022 May 12;25(4):1678–90. doi: 10.1111/hex.13511 (PMC9327825; doi:10.1111/hex.13511)
Supplement: Supplementary file 1 — Supporting information. [file HEX-25--s001.docx]

**Supplementary material: SCENARIOS FOR DIALOGUE GROUPS**

**Current Australian public health protocols for managing a measles outbreak**

Measles is highly contagious. It is spread via droplets (from coughing or sneezing) or direct contact with mucus or nose secretions from an infected person. A single person with measles will infect on average 17 other people if they are not vaccinated.

The infectious period is from roughly 4 days before to 4 days after the onset of the rash. The incubation period is from 7—18 days.

Those most susceptible to severe disease include:

- Immunocompromised people (e.g. people with leukaemia, lymphoma or AIDS)
- Malnourished children
- Children younger than 5 and adults over 20, who tend to have more complications
- Pregnant women (measles infection increases risk of premature labour, spontaneous abortion and low birth weight infants).

Susceptible people are more likely to experience complications such as ear infections, diarrhoea and pneumonia, and may need to go to hospital. About 1/1000 people with measles develops encephalitis (swelling of the brain).

In areas with low levels of vaccination, measles outbreaks tend to last longer; the lower the level of vaccination the longer this tends to be.

People who have or are suspected to have measles (called “cases”) are asked:

- not to attend school, early childhood education and care services or work from onset of symptoms to 4 days after onset of rash;
- to avoid contact with susceptible people (especially kids <1 year old, immunocompromised people); and
- to provide details of anyone they have been in contact with in the previous week.

Those contacts are asked:

- to stay away from school, early childhood education and care services, healthcare, and other settings with high risk contacts;
- to watch for signs and symptoms of measles; and
- to call ahead if they’re visiting the doctor to avoid infecting others.

They will also be offered vaccination if they are not vaccinated.

In situations where cases are confirmed, public heath units will offer vaccination, or post-exposure prophylaxis with immunoglobulin.

Hospital Emergency Departments (EDs), medical practitioners, school principals and early childhood education and care services directors are asked to report new suspected cases promptly to the local public health unit.

The local public health unit must report every case and suspected case of measles to the Commonwealth Department of Health.

**Introducing Amelia**

Amelia is 32 and has a one year old boy, Noah, and a five year old girl, Lily. Lily received the usual vaccination schedule up to 6 months. Soon after the 6 month shot she developed new tummy trouble that hung around for months, and went from being a great sleeper to hardly sleeping at all. After this, Amelia started reading and talking with her friends about vaccination, and decided to discontinue vaccinations for Lily. She didn’t vaccinate Noah at all.

Amelia and her husband Adam moved to a regional area before they got pregnant because they wanted a slower, less toxic lifestyle for themselves and their new family. Amelia would love to be a full-time mum to her kids, but has to work part time to provide enough income for the family.

Noah attends a parent-organised playgroup for unvaccinated kids two mornings a week. Lily attends the one local public school, where about 70% of the students are fully vaccinated. Amelia knows that several of her friends partially vaccinate their children or don’t vaccinate at all. But Amelia avoids talking about vaccination in her social groups because of worries about it causing tension.

**Introducing Glenn**

Glenn, 53, is the senior public health officer responsible for health protection in Amelia’s local area. Glenn grew up in the area. He went to the city to do a Master of Public Health in the mid-1990s and worked there with the health department for a few years. When a job came up managing the public health unit in his hometown, he decided with his partner and their two kids to return to the region. Glenn is ultimately responsible in the local area for population health, including preventing and managing serious infectious diseases across the region. This includes day-to-day work (like planning, monitoring and community engagement) as well as special responsibilities in outbreaks: for example, outbreaks of food poisoning, swine or bird flu, Hendra virus, or measles. In these situations, Glenn has special responsibilities to coordinate the response in his area, act upon special provisions in the public health act, and to report to and take direction from state authorities.

**SCENARIO BASED QUESTIONS**

**Scenario 1**

One Wednesday, Glenn receives a report from a local GP of a suspected case measles in a local primary school child. By Friday, five kids from the school have a confirmed measles diagnosis, three from one class and one each from two other classes. Glenn has to decide what actions to take to prevent further spread to the school and general community.

**Just pause for a minute and think about that scenario. What do you think Glenn would or should decide to do?**

On Friday afternoon, the principal sends a text and email to all parents and a note home with all students advising that there has been a measles outbreak at the school. One of the children with measles is in Lily’s class, so they get a separate special text and email about that, letting them know that the public health unit will be in touch shortly.

Glenn’s team talks to about 70 parents on Friday afternoon and evening and Saturday morning, including Amelia. The caller advises that Lily may have contracted measles at school, and asks whether she is vaccinated (Amelia says no).

The public health unit advises that because Lily is not vaccinated, she is considered to be a susceptible contact. They ask Amelia to keep Lily at home from school and observe her closely for fever, conjunctivitis, runny nose, cough or a rash, and call their GP immediately if these symptoms develop. They also ask Amelia to keep away from hospitals and medical centres, and to particularly avoid immunocompromised people, babies and pregnant women, and non-vaccinated children under 5 or adults over 20. They advise that they will call regularly. Once no more cases of measles appear at the school, they will count 14 days, then Lily and all of the other unvaccinated kids will be able to go back to school.

**Just pause for a minute and think about that scenario. What do you think Amelia and Adam would or should decide to do?**

**How reasonable do you think it is for Amelia and Adam to be asked to keep Lily at home?**

**What do you think Amelia and Adam should do about Noah?**

Scenario 2

Glenn’s team need to decide what other measures to put in place over the weekend. They have learned that the six kids with confirmed measles have recently visited a number of places where there was close contact with other kids for a long period (including music groups, dance classes, Sunday school, kids’ gym and yoga, a cinema and a birthday party). He has to decide how many of these contacts to trace.

Glenn knows that the National Guidelines for Public Health Units also suggest that he consider holding a vaccination clinic at the school (for kids whose parents consent to vaccination) and alerting the local media. He also needs to think about how to implement the recommended quarantine of exposed, unvaccinated kids.

**What do you think Glenn should do? What influences your thinking here?**

Glenn sends the Principal a list of kids whose parents have been asked to keep them at home. He organises for a school vaccination clinic on Tuesday morning, and send the Principal some background information.

Glenn checks the local and school Facebook pages repeatedly over the weekend. On the School’s page, the Principal has asked parents to keep their kids at home if asked, and has told them about the clinic, asking parents to look for a letter and consent note in their child’s bag on Monday afternoon.

Some parents have commented positively on the post, thanking the Principal for intervening and offering the vaccinations. Others are angry, confronting non-vaccinating parents. Yet others are blaming the school for over-reacting to a normal minor childhood illness, or expressing fear that children might be vaccinated without consent from their parents.

Over the weekend, Glenn receives notification of ten more cases of measles.

On Monday morning he gets a call from the Principal: eight kids from the three measles classes have come to school; two have a fever and a runny nose and one seems to be getting a rash. She asks Glenn what he thinks she should do.

**What do you think Glenn and the principal should do now? What influences your thinking here?**

**What should parents of kids at the school do now?**

**Scenario 3**

Suppose we add one more feature to the scenario. Suppose that in the third grade in Lily’s school there is a child who has leukaemia.

**Does that change your thinking about what Amelia and Adam should do with Lily?**

**Does it change your thinking about what Glenn and the principal should do about the situation?**

**Scenario 4**

Amelia keeps Lily home as requested, and by Wednesday she is looking very unwell. A GP visit confirms that Lily almost certainly has measles.

The GP suggests some strategies for treating Lily’s symptoms, and asks Amelia to keep Lily isolated at home until at least 4 days after the rash develops, and to be extremely careful to keep visitors away.

The GP also asks whether Amelia might be willing to vaccinate Noah. She says she won’t put any pressure on her, but wanted to give her the option.

**Just pause for a minute and think about that scenario. What do you think Amelia and Adam would or should do now?**

**FINAL REFLECTIVE QUESTIONS**

1. In these scenarios, we have asked what parents, public health officials, principals and GPs should do. We have asked what members of a society owe one another.
   1. What do you think it is reasonable for society to expect of parents in relation to vaccination?
      1. [PROMPT: Remember this is about conscientious objection arguments and reciprocity arguments – that if you CO from vaccination you might owe something in return]
2. In this project, we will have an opportunity to interact with decision-makers about what different groups of participants said. If you could say one thing to representatives of the health system, what would it be?
